# Mendelian randomization with a binary exposure variable: interpretation and presentation of causal estimates

**DOI:** 10.1007/s10654-018-0424-6

**Published:** 2018-07-23

**Authors:** Stephen Burgess, Jeremy A. Labrecque

**Affiliations:** 1 MRC Biostatistics Unit, Cambridge Institute of Public Health, University of Cambridge, Robinson Way, Cambridge, CB2 0SR UK; 2 Department of Public Health and Primary Care, University of Cambridge, Cambridge, UK; 3 Department of Epidemiology, Erasmus MC, Rotterdam, Netherlands

**Keywords:** Mendelian randomization, Genetic epidemiology, Causal inference, Instrumental variable, Effect estimation

## Abstract

Mendelian randomization uses genetic variants to make causal inferences about a modifiable exposure. Subject to a genetic variant satisfying the instrumental variable assumptions, an association between the variant and outcome implies a causal effect of the exposure on the outcome. Complications arise with a binary exposure that is a dichotomization of a continuous risk factor (for example, hypertension is a dichotomization of blood pressure). This can lead to violation of the exclusion restriction assumption: the genetic variant can influence the outcome via the continuous risk factor even if the binary exposure does not change. Provided the instrumental variable assumptions are satisfied for the underlying continuous risk factor, causal inferences for the binary exposure are valid for the continuous risk factor. Causal estimates for the binary exposure assume the causal effect is a stepwise function at the point of dichotomization. Even then, estimation requires further parametric assumptions. Under monotonicity, the causal estimate represents the average causal effect in ‘compliers’, individuals for whom the binary exposure would be present if they have the genetic variant and absent otherwise. Unlike in randomized trials, genetic compliers are unlikely to be a large or representative subgroup of the population. Under homogeneity, the causal effect of the exposure on the outcome is assumed constant in all individuals; rarely a plausible assumption. We here provide methods for causal estimation with a binary exposure (although subject to all the above caveats). Mendelian randomization investigations with a dichotomized binary exposure should be conceptualized in terms of an underlying continuous variable.

Mendelian randomization is the use of genetic variants as instrumental variables to test for or estimate the causal effect of a risk factor (referred to here as an exposure) on an outcome using observational data [11, 12]. The primary objective of Mendelian randomization is to find modifiable exposures that are worthwhile therapeutic targets and can be intervened on to improve health outcomes. An instrumental variable must be associated with the exposure of interest (relevance), only affects the outcome through the exposure (exclusion restriction), and does not share any causes with the outcome (exchangeability). Recently, several Mendelian randomization studies have employed binary measures as the exposure variable. Examples include analyses assessing the causal effect of cannabis initiation on schizophrenia (and of schizophrenia on cannabis initiation) [14, 24], and of diabetes status on endometrial cancer [17]. In this short manuscript, we discuss issues relating to causal estimation in the Mendelian randomization setting with a binary exposure. For ease of presentation, we initially assume a single genetic variant is used as an instrumental variable; this restriction is later relaxed.

The intended primary audience of this manuscript is Mendelian randomization practitioners, and the aim of the manuscript is to communicate the practical consequences of these methodological issues for Mendelian randomization investigations. As such, we focus on methods and approaches that are likely to be the most relevant to scenarios that are common in applied practice. In particular, we focus on methods that can be performed using summarized data, which comprise genetic associations with the exposure estimated using regression methods, that are routinely reported by large consortia [9]. Although our focus is on practitioners, we also provide technical asides and references for methodologically-focused readers.

## Random assignment in a trial as a paradigm instrumental variable

Consider a double-blind, placebo-controlled randomized trial with two time-fixed treatment arms (referred to as treatment and control) and complete follow-up data. An intention-to-treat effect estimate is typically reported: the causal effect of allocation to treatment as opposed to control. When there is substantial non-compliance, investigators may be interested in testing whether the treatment itself has an effect on the outcome (as opposed to simply allocation to treatment), or in estimating the causal effect of the treatment itself. Testing for a treatment or ‘per-protocol’ effect can be achieved through the intention-to-treat analysis: unless random assignment somehow affects the outcome directly (e.g., because blinding is broken or a placebo effect is present), an association between treatment allocation and the outcome will only arise if the treatment has a causal effect on the outcome [13]. Estimating the average treatment effect in the full study population further requires additional homogeneity conditions [4, 15, 25]; sufficient conditions are linearity of the instrumental variable-exposure, instrumental variable-outcome and exposure–outcome relationships with no effect heterogeneity. Without additional conditions, only bounds for the average treatment effect are obtainable [5]. These bounds can also be used to assess the validity of a genetic variant as an instrumental variable [19, 21], although this approach is rarely informative in practice, and alternative ways of assessing instrument validity (such as understanding the biological role of the genetic variant, and assessing its associations with known confounders) are more likely to be fruitful in practice [10].

As an alternative to estimating the average treatment effect in the full population, investigators often estimate an effect in a subgroup of the population under a weaker assumption. Specifically, we consider the subgroup of the population consisting of ‘compliers’—individuals who would receive the treatment if allocated to treatment, and would not receive treatment if allocated to not receive treatment. The effect in this subgroup can be estimated under the assumption that there are no defiers—individuals who would only take treatment if randomly allocated not to do so, and who would not take treatment if allocated to take it [2]. This is known as the monotonicity assumption—allocation to taking the treatment can only increase the value of the exposure, not decrease it. This effect, which can be estimated using standard instrumental variable techniques, is known as the local average treatment effect (LATE) or the complier average causal effect (CACE) in the literature [27]. Of note, we cannot identify individual compliers as we cannot see individuals’ treatment levels under both levels of treatment allocation. However, it is possible to identify the proportion of the study population who are compliers, and to describe relative characteristics of the compliers compared to non-compliers using measured baseline covariates [3]. In well-designed randomized trials, compliers are likely to be common, and the assumption that there are no defiers is often considered reasonable.

## Who are the genetic ‘compliers’?

Monotonicity in the context of Mendelian randomization means that increasing the number of effect alleles for an individual can only increase the exposure from absent to present (or leave it constant), and can never decrease it. (We here define the effect allele as the exposure-increasing allele without loss of generality.) The analogue of ‘compliers’ in Mendelian randomization are individuals who would have the exposure present if they possess an exposure-increasing allele, but would not otherwise. As genetic variants tend to have small effects on phenotypic variables, such compliers are likely to be uncommon.

This means that the group of genetic compliers is not likely to be representative of the general population. Also, the group of compliers may well differ greatly between different study populations. As an example, folate deficiency has been hypothesized as a causal risk factor for coronary heart disease [16]. The complier population (and therefore the instrumental variable estimate) would differ greatly in a population where large numbers of people are borderline folate deficient compared with a population where relatively few people are folate deficient. (A similar problem would occur in randomized trials conducted in different populations.) The analogous assumption in Mendelian randomization to the ‘no defiers’ assumption is that increases in the genotype variable would lead to increases (or no change) in the exposure for all individuals in the population (or equivalently, decreases or no change in the exposure for all individuals) [15]. With a genetic variant that takes multiple values, the equivalent assumption is that the exposure is a non-decreasing (or non-increasing) function of the genetic variant. In this case (and in the case with multiple genetic variants), the instrumental variable estimate is a weighted average of LATEs [1].

In the context of RCTs, even if individual compliers cannot be identified, the subgroup of compliers may be of interest either because it represents a large or representative subgroup of the population, or due to patterns of non-compliance in the trial being anticipated to be repeated outside the trial setting. However, in Mendelian randomization, the subgroup of genetic ‘compliers’ is unlikely to represent those individuals in the population who would respond to a treatment that influences the target exposure, particularly if the treatment has a greater effect on the risk factor than the genetic variant. Hence, under the ‘no defiers’ assumption, the interpretation of a causal estimate in a Mendelian randomization investigation in which the instrumental variable assumptions are satisfied is that of an average causal effect in those individuals whose exposure status would vary depending on whether they have a particular genetic variant or not. We additionally note that the subgroup of genetic compliers would differ between genetic variants. This provides yet another reason why causal estimates based on different genetic variants may vary even if all the genetic variants are valid instruments.

## What is the true risk factor underlying the exposure?

The above interpretation assumes that the instrumental variable assumptions are satisfied. These assumptions imply that the only influence of the instrumental variable on the outcome is via the exposure—if the instrumental variable changes, but the exposure stays the same, then the outcome should not change. However, for most binary exposures used in Mendelian randomization investigations, there is an underlying continuous risk factor for which the binary variable is a dichotomization. As a simple example, the binary exposure hypertension is a dichotomization of the continuous risk factor blood pressure. In more complex examples, an underlying continuous latent variable can be hypothesized even if it cannot be measured, such as a continuous spectrum of sub-clinical mental health problems for the binary exposure schizophrenia.

If the binary exposure is a dichotomization of a continuous risk factor, then the instrumental variable assumptions are likely to be violated. For the example of hypertension, if elevated blood pressure is a causal risk factor for a particular outcome then genetic variants that are associated with blood pressure will be associated with the outcome even in a population where no-one suffers from clinically-defined hypertension. Hence, changes in the genetic variants will lead to increases in blood pressure and consequently to changes in the outcome even if the exposure status for hypertension remains fixed for all individuals in the population. An instrumental variable for a continuous exposure can only be an instrumental variable for the dichotomization of the exposure if the exposure–outcome causal relationship is a strict stepwise threshold at the point of dichotomization (in which case the dichotomized exposure is a representation of the true risk factor). However, provided that the instrumental variable assumptions are satisfied for the continuous risk factor, testing for an association with the outcome is still a valid test of the causal null hypothesis for the binary exposure.

There are two main consequences of this. First, such a Mendelian randomization study should be conceptualized as an investigation into the (possibly latent) underlying continuous risk factor, rather than the binary dichotomization of this variable. At minimum, the instrumental variable assumptions should be assessed with the continuous risk factor in mind. Second, a causal estimate from a Mendelian randomization investigation with a dichotomized binary exposure does not have a clear interpretation due to the binary exposure variable not capturing the true causal relationship. There are several reasons why a Mendelian randomization estimate may differ from the effect of an intervention even for a continuous exposure (for example, genetic variants have long-term influences acting from the beginning of life, whereas interventions are more short-term and are applied to mature individuals) [8, 22]. With a binary exposure, these concerns are even greater.

## Causal estimation with a binary exposure

Because of these issues, several authors have recommended that Mendelian randomization investigations should only test the causal null hypothesis, rather than attempt to calculate a causal estimate [13, 23]. With a single genetic variant, this can be achieved by testing for an association between the variant and the outcome. With multiple genetic variants, the most efficient test of the causal null hypothesis is achieved by the IV estimate (under the homogeneity assumptions, the two-stage least squares estimate, or equivalently the inverse-variance weighted estimate, is the optimally efficient combination of the instruments for testing for a causal effect [26])—hence we may want to perform IV estimation to test the causal null even if the IV estimate is regarded as a test statistic and does not have a clear interpretation as a causal effect.

Suppose that we want to calculate a causal effect with a binary exposure, under the assumption that the exposure has a stepwise effect on the outcome (that is, it truly is a dichotomous exposure). This may be because we truly believe in the homogeneity assumptions, or we truly believe in the monotonicity assumption and regard the genetic compliers as a worthwhile subgroup of the population in which to estimate an average causal effect. Or, more likely, because a causal effect estimate is required for pragmatic reasons, such as to perform a power calculation or to inform policymakers of the expected impact of intervention on the exposure. Other reasons for estimating a causal parameter include efficient testing of the causal null hypothesis with multiple candidate instrumental variables, and using a robust method with multiple genetic variants (such as the MR-Egger method [6] or weighted median method [7]—these methods make weaker assumptions, not requiring all genetic variants to satisfy the instrumental variable assumptions). If the binary exposure is a dichotomization of a continuous risk factor, then power calculations are likely to be conservative, as the effect of the genetic variant on the outcome will not be fully captured by the binary exposure.

Two options for causal estimation are: (1) estimating the effect on the outcome per (say) 1% absolute increase in the probability of the exposure; (2) estimating the effect on the outcome per (say) doubling of the probability (or odds) of the exposure. We concentrate on estimation methods based on regression (usually linear or logistic) for several reasons. First, often researchers perform their analyses using summarized association estimates—beta-coefficients from regression analyses of the exposure and outcome on a genetic variant—and do not have access to individual-level data. These beta-coefficients represent the average change in the trait (exposure or outcome) per additional copy of the effect allele. Secondly, these approaches result in causal estimates with a simple and relevant interpretation, and which can be compared to estimates in the literature from other analytical approaches. Thirdly, often there are technical restrictions on the data analysis—for example, it may be necessary to fit a mixed model to account for relatedness between individuals, to adjust for several principal components of ancestry, or to provide a coordinated approach to analysis across different datasets. These restrictions are easiest to accommodate in a regression framework. These estimation procedures require strict linearity and homogeneity assumptions; full details are available elsewhere [13, 15]. The parametric assumptions for these two options are mutually incompatible. Additionally, regression coefficients will generally be variation dependent on the baseline risk, a nuisance parameter [20]. If individual-level data are available, then alternative approaches to estimation can be taken [4, 25].

If the genetic associations with the exposure are estimated using linear regression, then they represent absolute changes in the prevalence of the exposure. This enables estimation of the causal effect of an intervention in the prevalence of the exposure on an absolute scale. It is sensible to scale the causal effect to consider a modest increase in the prevalence of the exposure (say a 1% or a 10% increase), as a unit increase would represent the average causal effect of a population intervention from 0% prevalence of the exposure to 100% prevalence—an unrealistic intervention in practice. However, absolute associations with a binary variable do not make sense in case-control settings (where cases are those with the exposure), as they depend on the ratio of cases to controls chosen by the investigator.

If the genetic associations with the exposure are estimated using logistic regression, then they represent log odds ratios. The causal estimate would then represent the change in the outcome per unit change in the exposure on the log odds scale. A unit increase in the log odds of a variable corresponds to a 2.72 ($= \exp 1$)-fold multiplicative increase in the odds of the variable. If the exposure is rare then the odds of the exposure is approximately equal to the probability of the exposure. The causal estimate represents the average change in the outcome per 2.72-fold increase in the prevalence of the exposure (for example, an increase in the exposure prevalence from 1 to 2.72%). It may be more interpretable to think instead about the average change in the outcome per doubling (2-fold increase) in the prevalence of the exposure. This can be obtained by multiplying the causal estimate by 0.693 ($= \log _e 2$).

## Discussion

**Fig. 1:**
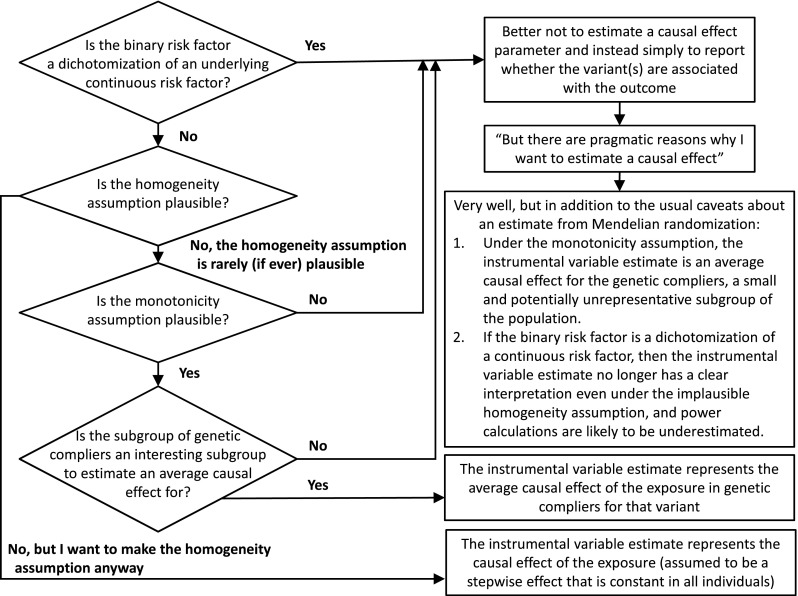
Flow diagram illustrating the steps needed to consider when considering whether to estimate a parameter in a Mendelian randomization investigation or not with a binary risk factor

In this short manuscript, we have discussed statistical issues for Mendelian randomization with a binary exposure. A summary of the arguments made in the paper is provided as Fig. 1. Under the more plausible assumption of monotonicity, the estimate from a Mendelian randomization study with a binary exposure represents the average causal effect in ‘compliers’; the subgroup of individuals for whom the presence or absence of the genetic variant used as an instrument determines whether individuals have the exposure present or not. Under the less plausible assumption of homogeneity, the estimate of the causal effect only makes sense if the effect of the exposure on the outcome has a strict stepwise form—only changes in whether the binary exposure is present or absent will affect the outcome. If the binary exposure is a dichotomization of a continuous variable, then the causal estimate does not have a clear interpretation. In such a case, causal inferences will only be valid provided that the instrumental variable assumptions are satisfied for the continuous risk factor—in particular, if the effect of the genetic variant on the outcome is completely mediated via the continuous risk factor. However, as the effect of the genetic variant on the outcome is not completely mediated via the binary exposure, power calculations are likely to be conservative. Additionally, no particular significance should be assigned to the point of dichotomization of a continuous risk factor—if a Mendelian randomization investigation suggests that hypertension affects a disease outcome, clinical interventions should not focus specifically on lowering blood pressure above a certain cutpoint, or on individuals with blood pressure values close to that cutpoint.

We have concentrated here on issues that are specific to the use of a binary exposure variable. There are several other considerations in a Mendelian randomization investigation, such as choice of genetic variants, validity of the instrumental variable assumptions, measurement error in the exposure, and similarity between samples in a two-sample Mendelian randomization analysis (where the genetic associations with the exposure and the genetic associations with the outcome are estimated in different datasets). While classical measurement error in the exposure does not affect asymptotic estimates from instrumental variable analysis for valid instruments [18], it will affect estimates with a dichotomized exposure variable, as measurement error in the continuous risk factor will lead to misclassification of the binary exposure and hence attenuation of the genetic association with the exposure (but not in the genetic associations with the outcome). In a two-sample setting, consistent estimation for a dichotomized exposure requires the stepwise effect of the exposure to act at the same level of the exposure in both populations. Otherwise, all considerations for a Mendelian randomization analysis with a continuous exposure are similarly relevant for a Mendelian randomization analysis with a binary exposure.

In summary, applying Mendelian randomization with a binary exposure requires careful consideration. When the binary exposure is a dichotomization of an underlying continuous risk factor, causal assumptions should be assessed and causal inferences should be conceptualized with respect to the underlying continuous risk factor. Tests for causal effects may be achieved readily without using the exposure information, but estimation procedures for a binary exposure require strong assumptions that are unlikely to be biologically plausible in common Mendelian randomization settings.
